# Application of Nursing Process and Its Affecting Factors among Nurses Working in Mekelle Zone Hospitals, Northern Ethiopia

**DOI:** 10.1155/2014/675212

**Published:** 2014-02-06

**Authors:** Fisseha Hagos, Fessehaye Alemseged, Fikadu Balcha, Semarya Berhe, Alemseged Aregay

**Affiliations:** ^1^Department of Nursing, Sheba University College, Mekelle, Ethiopia; ^2^Jimma University, Jimma, Ethiopia; ^3^Department of Nursing, University of Mekelle, Ethiopia

## Abstract

*Background.* Nursing process is considered as appropriate method to explain the nursing essence, its scientific bases, technologies and humanist assumptions that encourage critical thinking and creativity, and permits solving problems in professional practice. *Objective.* To assess the application of nursing process and it's affecting factors in Mekelle Zone Hospitals. *Methods.* A cross sectional design employing quantitative and qualitative methods was conducted in Mekelle zone hospitals March 2011. Qualitative data was collected from14 head nurses of six hospitals and quantitative was collected from 200 nurses selected by simple random sampling technique from the six hospitals proportional to their size. SPSS version 16.1 and thematic analysis was used for quantitative and qualitative data respectively. *Results.* Majority 180 (90%) of the respondents have poor knowledge and 99.5% of the respondents have a positive attitude towards the nursing process. All of the respondents said that they did not use the nursing process during provision of care to their patients at the time of the study. Majority (75%) of the respondent said that the nurse to patient ratio was not optimal to apply the nursing process. *Conclusion and Recommendation.* The nursing process is not yet applied in all of the six hospitals. The finding revealed that the knowledge of nurses on the nursing process is not adequate to put it in to practice and high patient nurse ratio affects its application. The studied hospitals should consider the application of the nursing process critically by motivating nurses and monitor and evaluate its progress.

## 1. Background

The nursing process was originally adopted by the north American nursing profession from the general systems theory (GST) and quickly became a symbol of contemporary nursing as well as a professionalism nurse ideology [1].

The nursing process is considered the appropriate method to explain the nursing essence, its scientific bases, technologies, and humanist assumptions that encourage critical thinking and creativity and permits solving problems in professional practice. This method represents an attempt to evidence and understand nursing work focused on care as a reflective practice [2].

The nursing process is a widely accepted method and has been suggested as a scientific method to guide procedures and qualify nursing care. More recently, the process has been defined as a systematic and dynamic way to deliver nursing care, operating through five interrelated steps: assessment, diagnosis, planning, implementation, and evaluation [3].

According to current American and Canadian practice standards, nursing practice demands the efficient use of the nursing process and professional participation in activities that contribute to the permanent development of knowledge about this methodology [3].

Nurses are the largest group of health professionals in all countries. Nursing quality is closely related to a healthcare system's effectiveness. In order to achieve quality of health care service, quality of nursing care is the key element and to fill this demand application of the nursing process has a significant role, but, in practice, application of the nursing process is not well developed. Nursing process is also the base of nursing researches and different researches undertaken on the documents of nursing process and nursing diagnosis, but due to application of nursing process, nursing research is still not well developed globally. Not all of the nurses, of course, were convinced of the appropriateness of a scientific nursing process basis for nursing research and practice. Nurses with greater levels of expertise are less likely to use the nursing process as a complete system or package for care, relying more on intuition and/or experienced clinical judgments which might also worsen the problem [1].

Implementing a new methodology to guide nursing care delivery implies facing a series of challenges, which requires a priori acknowledgement of the institution's and the nursing team's possibilities and limits [4].

Implementation of the nursing process in practical nursing can be achieved in accordance with the principles of action research. A prerequisite of the action research is knowledge of the basic principles and the component areas of the nursing process and knowledge of the opportunities for applying the nursing process to practical work, but knowledge of nurses on this process is not studied yet [5].

Effective implementation of the nursing process leads to improved quality of care and stimulates the construction of theoretical and scientific knowledge based on the best clinical practice. In Ethiopia, health services are limited and of poor quality [6]. The quality of nursing care is also perceived as poor. To improve the quality of nursing care, the basic thing is application of the nursing process and for its implementation, the government has been investing on educating students in different educational status at school level based on the nursing curriculum. But the application of this knowledge in practical setup is not well known yet.

Thus, undertaking study related to nursing process application and its affecting factors so as to identify knowledge and/or skill gaps is mandatory. And the result of this study may contribute some importance for policy makers and health care planners in application of nursing process which will have a positive outcome in quality of nursing care provided in Mekelle Zone in particular and for the country in general. As it was known there is no literature in the country, which shows the implementation level of nursing process and its affecting factors in Ethiopia, Mekelle Zone. Therefore, this study aimed to assess the application of nursing process and affecting factors. Further, the result of the study can be used as a baseline data for further related studies. 

## 2. Methods

### 2.1. Study Settings

The study was carried out in Mekelle the capital city of Tigray region, which is 783 kilometer far from Addis Ababa city to north direction. Mekelle Zone has seven hospitals which contains one teaching (referral) hospital, two public (governmental) hospitals, one defense hospital, and three private hospitals. Total numbers of nurses found in the seven hospitals were 377. The study was conducted on March 2011.

### 2.2. Study Design, Participants, and Sampling Procedure

A cross sectional quantitative and qualitative study designs were employed and study participants were selected, 210 nurses from the seven hospitals found in Mekelle Zone for quantitative study and all head nurses for qualitative study. Sample size for the quantitative study was calculated using single proportion based on the prevalence off the proper application of nursing process, but as there are no studies showing the prevalence of application of the nursing process, a population proportion of 50% was used to maximize the sample size.

Using the correction formula, final sample size becomes 191 and including 10% nonresponse rate, the final sample size is 210. The number of nurses from each hospital was obtained by proportional allocation with 81 nurses from Ayder, 47 nurses from Mekelle, 25 nurses from Quiha, 47 nurses from Semen EZ, 4 nurses from Markos, and 3 nurses from Meskerem hospitals; the remaining one private hospital was not included in the study because it was closed during the data collection period. Fourteen head nurses from six hospitals of Mekelle Zone were involved in the qualitative study; the number of head nurses was determined by saturation of the required data.

Simple random sampling technique was used to select nurses after proportional allocation of sample size to each hospital; lists of nurses were received from personnel office of each hospital and then a lottery method was employed. Fourteen head nurses who were selected purposively from the six zonal hospitals were participating in the in-depth interview purposely. The number of key informants from the six hospitals was determined by saturation of required information.

### 2.3. Data Management and Analysis

For quantitative data, after checking the completeness, missing values, and coding of questionnaires, data were entered to computer and processed and analyzed using SPSS version 16.0. Pretest was conducted on 10% of the study population in Addis Ababa in Betel and Black Lion hospitals.

The data were summarized and described using descriptive statistics and binary logistic regression was used to determine the relationship of sociodemographic characteristics with knowledge and attitude of nurses on nursing process. Then, finally, data were presented in tables, graphs frequency percentage of different variables.

For qualitative data, data from in-depth interview of participants were analyzed by thematic analysis technique and described each theme descriptively.

### 2.4. Ethical Considerations

Letter of ethical clearance was obtained from Research Ethical review board of Jimma University. Letter for cooperation from each hospital was obtained; verbal consent was obtained from each nurse for participation in the study. Privacy and confidentiality were ensured during the interview, and name and address of the interviewee were not recorded in the questionnaire.

## 3. Result

In this study both quantitative and qualitative data collection methods have been employed. Out of the 210 questionnaires distributed among the study populations, 200 were filled completely and returned making the response rate 95.2%. In-depth interview involving 14 head nurses of six hospitals was also employed to gather data qualitatively.

### 3.1. Quantitative and Qualitative Findings

#### 3.1.1. Demographic Results

From the 200 participants the majority 123 (61.5%) were female while 48 (24.0%) were within the age group 35–39 years. One hundred thirty-one (65.5%) of the respondents were diploma nurses. With respect to their years of experience, 62 (31.0%) had up to four years of experience, while 11 (5.5%) of the respondents had work experience more than 25 years at the time of the study. The majority, 140 (70%), of the respondents acquired their educational award from government institutions and 61 (30.5%) of the respondents have been working at the medical ward at the time of the study (Table 1).

Out of the total 14 key informants, nine of them were females with age ranging from 24 to 42 years. The majority, 9 (64%), of them were BSc nurses; the rest 5 (36%) were diploma nurses. Medical and surgical wards contributed the majority (five each), and the others are 1 from OR, 1 from ART, 1 from gynecology, and 1 from pediatric wards involved, with a service year ranging from 2 to 22 years.

#### 3.1.2. Knowledge Related Results

Out of the total respondents, 181 (90.5%) reported that they have heard about nursing process, but 61 (30.5%) failed to mention the steps of nursing process effectively. For example, 71 (35.5%), 67 (33.5%), and 55 (27.5%), respectively, failed to mention evaluation, implementation, and the nursing diagnosis steps.

**Figure 1:**
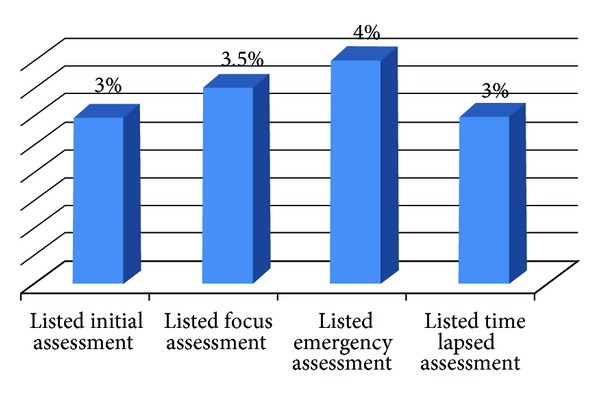
The distribution of nurses of Mekelle Zone Hospitals according to their knowledge on nursing assessment, Mekelle, northern Ethiopia, March 2011 (*n* = 200).

The respondents were required to mention the types/number of nursing assessments and the majority of them were unable to mention them. For instance, 194 (97%) failed to mention initial nursing assessment and 192 (96%) failed to mention emergency nursing assessment (Figure 1).

The respondents were required to mention the number of nursing diagnoses and the majority of them were unable to mention them correctly, 8 (4%) of them responded one, 85 (42.5%) responded two, 6 (3%) responded three, 2 (1%) responded four, only 5 (2.5%) responded the correct answer five, and the rest 94 (47%) failed to respond to the question.

**Figure 2:**
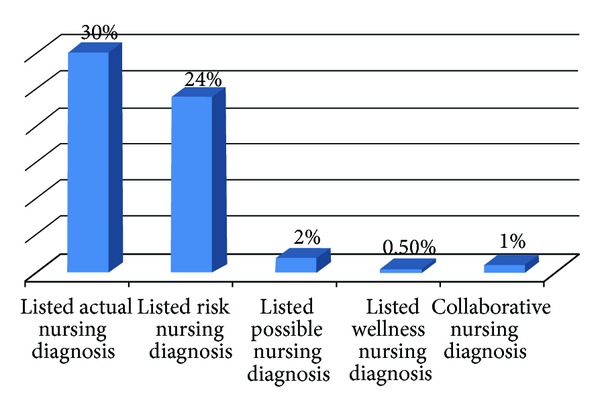
Distribution of nurses of Mekelle Zone Hospitals according to their awareness on the type of nursing diagnosis, northern Ethiopia, March 2011.

The study finding showed most of nurses have no knowledge on the nursing diagnosis; 60 (30%) of respondents mentioned actual nursing diagnosis; 48 (24%) of respondents mentioned risk nursing diagnosis; and 4 (2%) of respondents mentioned possible nursing diagnosis (Figure 2).

The study findings showed that regarding the nursing planning, the majority of them did not know its components. For instance, 192 (96%) failed to list developing nursing care plan, 187 (93.5%) failed to mention intervention, and 163 (81.5%) failed to mention even setting priorities.

The respondents did not have an appreciable awareness about implementation and evaluation, in which 76.5% did not answer the question about implementation of nursing care plan while 65.5% failed to answer the question regarding evaluation.

**Figure 3:**
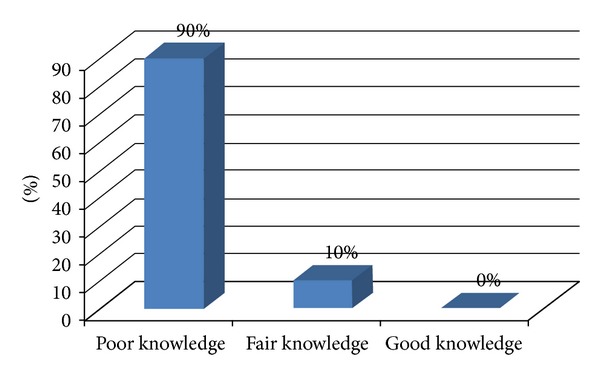
The frequency distribution of the respondents according to their knowledge status on nursing process in Mekelle Zone hospitals, northern Ethiopia, March 2011.

Regarding the overall knowledge of nurses on nursing process, the majority, 180 (90%), of them have poor knowledge while 20 (10%) of them have fair knowledge (Figure 3).

From the in-depth interview, it was found that nurses have no adequate knowledge to implement nursing process which may be due to variety of reasons. The majority of the key informants said that it is quite unlikely to put the nursing process in practice depending on the current knowledge of the nurses on the nursing process. For instance, one of the key informants said “*I do not believe that nurses have enough knowledge about the nursing process*.” According to claims made by some of the participants, inability to apply what the nurses have learnt at school was one of the reasons why nurses did lack knowledge to implement the nursing process. One key informant said “*nurses have no knowledge since as the time goes by we lose knowledge because what we have learnt about the nursing process and what we are doing now are quite different*.” Another key informant said “*we forgot the knowledge about nursing process because even if we learnt it at school we haven't been able to put it in practice*.”

From the educational level point of view, the key informants made it known that BSc nurses may have better knowledge to apply the nursing process. One key informant said “*I think BSc nurses have better knowledge than diploma nurses to apply the nursing process.*” The other key informant said “*I think BSc nurses have better knowledge in nursing process than diploma nurses because the nursing process is included in the curriculum of BSc nursing*.” Another key informant attempted to compare the knowledge of past and current nurses on the nursing process, saying “*the previous nurses had not had enough knowledge on nursing process as it was not included in their curriculum, but the current do have*.” In some cases, the problem may not be only knowledge on nursing process. Language barrier may be one of the factors hindering the application of nursing process, as one key informant indicated “*currently our diploma nurses have basic language problems. In fact they do not even know the five steps of nursing process*.”

#### 3.1.3. Attitude of Nurses on Nursing Process

Around 190 (95%) of the respondents reported that they either strongly agreed or agreed that the aim of the nursing process is appreciable, at same time 177 (88.5%) either strongly agreed or agreed that they were convinced that nursing process would work if applied in patient care. Slightly above 60% of the respondents showed their disagreement that the nursing process should be used only by BSc and above nurses. Seventy-nine percent of the respondents either strongly agreed or agreed that the nursing process works well in practice. Slightly above half of the respondents reported that there were time constraints to apply the nursing process; at same time 43% said that the nursing process is time wastage. In contrast, 81.5% indicated their readiness to apply the nursing process. Around 88% of the respondents said that the nursing process enables to provide quality nursing care, while 12.5% said that patients may not like to be cared for using nursing process. Around 55% of the respondents also either disagreed or strongly disagreed to the statement “nurse staffs have no willingness to apply the nursing process” (Table 2).

The mean score of the attitudes of nurses is 71.81 with a minimum score of 38 and maximum score of 92 out of 100.

Almost all of the respondents, 199 (99.5%) of the respondents, have scored above 50%; this indicates they have a positive attitude towards the nursing process.

All head nurses participated in this qualitative study agreed that nursing process will improve patient care. One key informant said “*I think if patient get service based on the nursing process, it is quite better.*” Another participant said “*the nursing process is quite important because it helps us to identify the patients' problems every hour and every day*.” Another key informant also strengthened this idea by saying “*Yes it improves service provided because it is the science that simplify the way to identify patients' problems*.”

The participants were also required to express their attitudes towards the type of health institution in which the nursing process needs to be applied. There are two types of attitudes reflected towards the setting in which nursing process can be applied. The first attitude is that the nursing process should be applied where there is a patient admission. Five participants said “*It is better to apply it in hospitals and admission areas.*” But some of the participants believed that the nursing process can be applied in every setting where patients and nurses are available. They said “*We can apply the nursing process wherever we want as long as nurses and patients are available*.”

Except for one participant, all the others believed that the nursing process can be applied by all nurses including diploma nurses after provision of training to them. One participant said “*I think nurses starting from diploma can apply the nursing process.*” Another participant said “*If they get better training any nurse can apply it.*” Again another participant said “*Since the number of nurses in Ethiopia is very low, all nurses must apply the nursing process after providing training to them*.” But one participant reflected his/her opinion indicating the nursing process is better applied by BSc nurses. He/she said “*as to me, BSc nurses can better apply the nursing process*.”

The participants were also asked to guess what the attitude of the other nurses would be towards the nursing process. Their responses were quite variable. Some of them said nurses have good attitude towards the nursing process. One participant, for instance, said “*they have good attitude*.*”* Another participant said *“We can say it is good*.” Some other participants said that nurses have good attitude towards nursing process but with some preconditions. For instance, one participant said “*nurses like the nursing process, and want to apply it but the number of nurses is not sufficient enough to the number of patients as a result nurses were not motivated to apply it*.” Another participant said “*the attitude of nurses is good if it is supported by training*.” The other participant said “*the nurses have better attitude to apply it but our hospital serves patients coming from four regions. Due to this, the nurses became exhausted because of high work load*.”

The other participants said nurses do not give attention to nursing process due to different reasons. One of them said “*due to lack of knowledge they do not give attention*,” and the other one said “nurses do not give attention towards nursing process because they give care traditionally.”

#### 3.1.4. Practice Related Results

All of the respondents said that they did not follow the scientific ways of application of the nursing process during provision of care to their patients at the time of the study (Table 3).

With regard to the application of the nursing process, all participants unanimously stated that the nursing process was not applied in patient care. One of the key informants said “*there is a rule indicating the application of the nursing process in every patient care within 24 hours of admission, but it is only a paper value”.* The other participant said “*till now we have not yet started applying the nursing process.*”

#### 3.1.5. Relationship of Sociodemographic Characteristics with Knowledge and Attitude

The knowledge of the respondents on nursing process has a significant relationship with their educational status. Compared to the knowledge of diploma nurses, the knowledge of BSc nurses on nursing process is higher by about 11.5 times (*P* < 0.001). But the rest of the demographic characteristics have no statistically significant association with knowledge on nursing process (Table 4).

Association of attitudes of the respondents with the socio-demographic characteristics showed that none of them has statistically significant association in binary logistic regression; that is, the attitude of nurses towards the nursing process is not affected by the sociodemographic characteristics.

#### 3.1.6. Enabling and Reinforcing Factors for Implementation of the Nursing Process

Regarding the enabling and reinforcing factors, 94 (47%) said that the administrations of the hospitals were supportive in the application of the nursing process, while 51 (25.5%) of the respondents reported that allocation of resources for application of nursing process was adequate. The majority, 150 (75%), of the respondents said that the nurse to patient ratio was not optimal to apply the nursing process, whereas only 11 (10.5%) said that the salary and promotion were motivating for the application of the nursing process, despite that around 118 (59%) of the respondents claimed that their educational level was adequate to apply the nursing process (Table 5).

It is well known that one of the limitations of the nursing process is that it needs ample of resources especially time. Most of the key informants believe that most of the enabling and reinforcing factors such as time, resource, human power, and training were not adequate to apply nursing process. One of the key informants said “*The main obstacles for application of the nursing process are resource scarcity, time shortage and lack of adequate knowledge*.” The other key informant supported the previous one by saying “*There is shortage of material and human power. In addition to that there is also no motivation at all to apply the nursing process*.” Some of the key informants said that lack of training on application of the nursing process is one of the factors why nurses lack adequate knowledge to apply the nursing process. Another key informant said “*Lack of attention and training are factors contributing to the failure of applying the nursing process.*”

## 4. Discussion

The nursing process is a widely accepted method and has been suggested as a scientific method to guide procedures and qualify nursing care [5]. In Ethiopia, quality of health care was poor [7] and to improve the quality of health service, application of nursing process may contribute a lot.

### 4.1. Knowledge

The findings of this study indicated that knowledge is one of the most determinant factors for application of the nursing process. One hundred eighty (90%) of the respondents have scored below 50% on knowledge related questions. All the key informants indicated that nurses lack knowledge to apply the nursing process. The findings of this study agree with the findings of a study conducted in Mexico which indicated that there was a problem in application of the nursing process due to variations in what the nurses were taught at school and what they were applying at hospitals [4]. The result of this study also agreed with a study conducted in Brazil which indicated knowledge is one of several factors that interfere in the efficient implementation of the nursing process [8].

### 4.2. Attitude

According to both the qualitative and quantitative findings, nurses of the study sites have positive attitude towards the nursing process. One hundred ninety-nine (99.5%) of the respondents have positive attitudes. The qualitative finding also revealed that nurses have positive attitude towards the nursing process. Most of key informants they believe nurses are qualified to do nursing process. The problem was not related to attitude; rather it was mainly related to knowledge and enabling and reinforcing factors because they believe it qualifies nursing care; it strengths nurse patient relationships; and it increases competency of the nurses. The findings of this study almost consistence the findings of a study conducted in US in which the mean attitudes of nurses towards the nursing process was 73.57% [9], and that of this study's mean attitude of nurses towards the nursing process was 71.81% indicating attitude is not a determinant for application of the nursing process.

### 4.3. Application of the Nursing Process

Both the qualitative and quantitative findings indicated that the nursing process is not applied by following the scientific way in the hospitals. All of the 200 respondents reported that they did not apply any of the nursing process steps. This finding is lower than the findings of study conducted in Brazil in which, for instance, assessment was performed in 98.7% of cases; diagnosis was made in 90% of cases; and planning was made in 74.8% of cases [3]. It also varies with a finding of a study conducted in central Taiwan revealed that nurses generally followed the nursing process and charting sequence to complete care plans [10]. It also varies with a study done in Nigeria indicating the nurses implement the nursing process 40.37%, 13.76%, 43.12%, and 2.75% at the level of assessment, level of nursing diagnosis, nursing care plan, and evaluation, respectively [11]. The variation may be due to difference in the study sites, the progress of the nursing profession, resource and technological variations, government commitment, level of nursing practice, and lack of clear nursing standard.

The findings of the qualitative study also strengthened the findings of the quantitative findings, in which all the participants said that any of the nursing process steps was not applied in their hospitals. This finding agrees with findings of a qualitative study conducted in a Mexican hospital that indicated there was a problem of application of the nursing process [2].

### 4.4. Sociodemographic Relationship

Among the sociodemographic characteristics, educational status has a statically significant relationship with knowledge of nurses on nursing process that BSc nurses have better knowledgeable than Diploma nurses with *P* value < 0.001.

### 4.5. Enabling and Reinforcing Factors

The result of this study showed that most of the enabling and reinforcing factors did not motivate nurses to apply nursing process. For example, slightly less than half (47%) of the respondents said that the administration of the hospitals supported application of nursing process; 25.5% said the allocation of time was adequate to apply nursing process; 67.5% said there was time shortage; and 75% said there was imbalance between the nurse and patient ratio. The quantitative finding is also supported by the qualitative finding. For instance, one key informant said “*The main obstacles for application of the nursing process are resource scarcity, time shortage and lack of adequate knowledge*.” Another respondent said “*We have not started to apply it because one nurse serves for 45 patients.*”

Finding of this study agrees with study conducted in Brazil indicating factors such as lack of knowledge of the steps involved in the process, excessive number of tasks assigned to the nursing team can interfere with the efficient implementation of the nursing process [10]. When compared with a study done in Nigeria for 36 (32.7%) ad 34 (29%) responded lack of regular supply and no supply of nursing process material respectively indicated to be a factor affecting implementation of nursing process. This variation could be the economical difference of the two countries [11]. To apply the nursing process in practice it requires adequate time, nursing human power and materials.

## 5. Conclusion

The majority (90%) of the study participants were poorly knowledgeable about the nursing process. Almost all of study participants had positive attitude towards the nursing process. This seems that nurses' attitude towards the nursing process is not a factor affecting the application of nursing process. From the sociodemographic characteristics, only educational status has direct statistically significant relationship with the knowledge of nurses on nursing process. Participants reported that factors such as shortage of resources, lack of knowledge, high patient nurse ratio/work load, and lack of training and motivating factors such as salary affected the application of the nursing process. It is possible to recommend the hospitals as well as the nurses to seek means to upgrade the knowledge of their nurses on the nursing process and its implementation and the nurses to improve their knowledge on the nursing process application respectively. The government must reemphasize on the provision of adequate resources such as materials, nursing human power, and adequate salary for the professionals so that the nursing process may be applied.

## Figures and Tables

**Table 1:** The frequency distribution according to the respondents by their demographic characteristics in Mekelle Zone Hospitals, northern Ethiopia, March 2011 (*n* = 200).

Demographic characteristics	Number	Percent
Sex		
Male	77	38.5
Female	123	61.5
Age		
<20	3	1.5
20–24	33	16.5
25–29	45	22.5
30–34	37	18.5
35–39	48	24.0
40–44	21	10.5
45–49	10	5.0
≥50	3	1.5
Educational status		
Diploma	131	65.5
BSc	67	33.5
MSc	2	1.0
Years of experience		
0–4	62	31.0
5–9	51	25.5
10–14	29	14.5
15–19	28	14.0
20–24	19	9.5
≥25	11	5.5
Institutions from where educational award is obtained		
Government	140	70.0
Private	60	30.0
The name of the hospital which the respondents are working in		
Ayder	80	40.0
Mekelle	46	23.0
Quiha	24	12.0
Semen EZ	43	21.5
Markos	4	2.0
Meskerem	3	1.5
The unit in which the respondents are currently working in		
Medical	61	30.5
Surgical	54	27.0
Pediatric	22	11.0
Oby-gyne	19	9.5
ICU	10	5.0
Others	34	17.0
The position of the nurse		
Nurse officer	3	1.5
Head nurse	1	0.5
Staff nurse	196	98.0

**Table 2:** The attitudes of the respondents towards the nursing process in Mekelle Zone Hospitals, northern Ethiopia, March 2011 (*n* = 200).

Variables	Strongly agree		Agree		I do not know		Disagree		Strongly disagree	
	Number	%	Number	%	Number	%	Number	%	Number	%
I like the aim of nursing process	74	37.0	116	58.0	4	2.0	1	0.5	5	2.5
I am convinced the NP will work if applied in patient care	55	27.5	122	61.0	11	5.5	8	4.0	4	2.0
The nursing process is an elaborated Kardex system	24	12.0	77	38.5	42	21.0	50	25.0	7	3.5
The nursing process should be used by BSc and above nurses only	13	6.5	43	21.5	17	8.5	97	48.5	30	15.0
The nursing process works well in practice	48	24.0	110	55.0	18	9.0	13	6.5	11	5.5
The nursing process can be used in any settings	38	19.0	107	53.5	28	14.0	21	10.5	6	3.0
There is no enough time to apply NP during pt care	49	24.5	61	30.5	15	7.5	53	26.5	22	11.0
Nursing process is a waste of time	35	17.5	51	25.5	25	12.5	58	29.0	31	15.5
I am ready for the application of nursing process	34	17.0	129	64.5	19	9.5	9	4.5	9	4.5
The Kardex system of nursing record is unsatisfactory	20	10.0	97	48.5	34	17.0	28	14.0	21	10.5
The NP simplifies the awareness of pt needs	45	22.5	123	61.5	14	7.0	12	6.0	6	3.0
Priorities of care are easy to identify using NP	54	27.0	124	62.0	15	7.5	7	3.5	0	0
I am fed up with hearing about the nursing process	26	13.0	76	38.0	41	20.5	49	24.5	8	4.0
The nursing process involves too much of paper work	18	9.0	61	30.5	23	11.5	74	37.0	24	12.0
NP enable nurses to provide quality of nursing care to pts	54	27.0	122	61.0	10	5.0	10	5.0	4	2.0
I am willing to apply nursing process during pt care	51	25.5	123	61.5	13	6.5	7	3.5	6	3.0
I think introduction of NP will cause a problem	6	3.0	25	12.5	45	22.5	94	47.0	30	15.0
I think pts will not like to be cared for using the NP	7	3.5	18	9.0	45	22.5	102	51.0	28	14.0
I think the nursing staff have no willingness to apply NP	13	6.5	38	19.0	38	19.0	90	45.0	21	10.5
I think the staff will never accept the nursing process	20	10.0	25	12.5	31	15.5	99	49.5	25	12.5

**Table 3:** Description the application of nursing process by the nurses of Mekelle Zone Hospitals, northern Ethiopia, March 2011 (*n* = 200).

Variables	Yes		No	
	Number	%	Number	%
Do you follow the steps of nursing process during provision of care?	0	0	200	100.0
Does data collection take place during the assessment phase?	0	0	200	100.0
Have you developed nursing diagnosis from your assessment?				
Actual	0	0	200	100.0
Risk	0	0	200	100.0
Possible	0	0	200	100
Wellness	0	0	200	100
Collaborative	0	0	200	100
Have you been preparing care plan based on your diagnosis?	0	0	200	100
Have you been implementing the care plan you have developed?	0	0	200	100
Have you been evaluating the effectiveness of your intervention?	0	0	200	100
Have you been documenting your nursing intervention?	0	0	200	100

**Table 4:** Association of knowledge with sociodemographic characteristics on binary logistic regression in Mekelle Zone Hospitals, northern Ethiopia, March 2011.

	*B*	Significance	COR	95.0% C.I.	
				Lower	Upper
Educational status		0.000			
BSc	2.442	0.000	11.493	3.808	34.685
MSc	3.041	0.089	20.935	0.628	697.644
Diploma		1			
Work experience		0.454			
5–9 years	−0.120	0.858	0.887	0.239	3.299
10–14 years	−0.711	0.564	0.491	0.044	5.505
15–19 years	−2.244	0.113	0.106	0.007	1.700
≥20 years	−0.360	0.800	0.698	0.043	11.359
0–4 years		1			
Age of respondent		0.387			
25–29 years	0.345	0.602	1.411	0.386	5.162
30–34 years	−0.887	0.515	0.412	0 .029	5.942
35–39 years	1.357	0.312	3.886	0.280	53.889
40–44 years	0.174	0.922	1.190	0.037	38.087
≥45 years	−18.528	0.999	0.000	0.000	.
20–24 years		1			
Type of hospital		0.287			
Public	−1.429	0.157	0.239	0.033	1.737
Defense	−1.956	0.074	0.141	0.017	1.210
Private	−1.925	0.115	0.146	0.013	1.596
Teaching		1			
Certificate awarded	0.095	0.848	1.099	0.418	2.888
Private	−1.231	0.261	0.292		
Government		1			

**Table 5:** Enabling and reinforcing factors for the application of nursing process by the nurses of Mekelle Zone Hospitals, northern Ethiopia, March 2011 (*n* = 200).

Variables	Yes		No		I do not know	
	No.	%	No.	%	No.	%
Does the hospital administration support the application of NP?	94	47.0	73	36.5	33	16.5
Is the allocation of resources for application of NP adequate?	51	25.5	128	64.0	21	10.5
Is allocated time sufficient to apply the nursing process?	52	26.0	135	67.5	13	6.5
Is the nurse/patient ratio optimal to apply the nursing process?	38	19.0	150	75.0	12	6.0
Is appreciating feedback available for application of NP?	43	21.5	141	70.5	16	8.0
Are there monitoring and evaluation for application of NP?	49	24.5	140	70.0	11	5.5
Are the salary and promotion motivating for application of NP?	21	10.5	173	86.5	6	3.0
Have you ever seen other nurses applying the NP?	40	20.0	148	74.0	12	6.0
Have you got on job training on nursing process?	42	21.0	150	75.0	8	4.0
Is your educational level adequate to apply NP?	118	59.0	78	39.0	4	2.0

## Acronyms

ART: Antiretroviral,
BSN: Bachelor of science in nursing
GST: General system theory
ICU: Intensive care unit
NP: Nursing process
SMART: Specific, measurable, attainable, realistic, and time-bound.
